# New perspectives for fascioliasis in Upper Egypt’s new endemic region: Sociodemographic characteristics and phylogenetic analysis of *Fasciola* in humans, animals, and lymnaeid vectors

**DOI:** 10.1371/journal.pntd.0011000

**Published:** 2022-12-28

**Authors:** Alzahraa Abdelraouf Ahmad, Haidi Karam-Allah Ramadan, Waleed Attia Hassan, Mohammed Ageeli Hakami, Enas Abdelhameed Mahmoud Huseein, Sara Abdel-Aal Mohamed, Adnan Ahmed Mohamed, Nahed Ahmed Elossily

**Affiliations:** 1 Faculty of Medicine, Department of Medical Parasitology, Assiut University, Assiut, Egypt; 2 Faculty of Medicine, Department of Tropical Medicine and Gastroenterology, Assiut University, Egypt; 3 Department of Clinical Laboratory Sciences, College of Applied Medical Sciences, Al-Quwayiyah, Shaqra University, Riyadh, Saudi Arabia; 4 Faculty of Veterinary medicine, Department of Parasitology, Assiut University, Assiut, Egypt; University of Liverpool, UNITED KINGDOM

## Abstract

**Background:**

Fascioliasis is a significant vector-borne disease that has emerged in numerous tropical and subtropical countries causing severe health problems. Egypt is one of the fascioliasis endemic regions; however, the current situation in Upper Egypt is understudied, with only sporadic human cases or outbreaks. This study aims to highlight the sociodemographic characteristics of human fascioliasis in a newly emerged endemic area in Upper Egypt, along with risk factors analysis and the molecular characteristics of the fasciolid population in humans, animals, and lymnaeid snails.

**Methodology/Principal findings:**

The study reported *Fasciola* infection in patients and their close relatives by analyzing the risk of human infection. Morphological and molecular characterization was performed on lymnaeid snails. Multigene sequencing was also used to characterize fasciolids from human cases, cattle, and pooled snail samples. The study identified asymptomatic *Fasciola* infection among family members and identified the presence of peridomestic animals as a significant risk factor for infection. This is the first genetic evidence that *Radix auricularia* exists as the snail intermediate host in Egypt.

**Conclusions/Significance:**

This study revealed that Assiut Governorate in Upper Egypt is a high-risk area for human fascioliasis that requires additional control measures. *Fasciola hepatica* was the main causative agent infecting humans and snail vectors in this newly emerged endemic area. In addition, this is the first report of *R*. *auricularia* as the snail intermediate host transmitting fascioliasis in Upper Egypt. Further research is required to clarify the widespread distribution of *Fasciola* in Egypt’s various animal hosts. This provides insight into the mode of transmission, epidemiological criteria, and genetic diversity of fasciolid populations in Upper Egypt.\

## Author summary

Human fascioliasis is a freshwater snail-transmitted disease that is widely distributed in many tropical and subtropical countries including Egypt. Recently, Upper Egypt reported sporadic cases of human fascioliasis and a few outbreaks establishing a new endemic area. This study was conducted to get information on the sociodemographic characteristics of the infected patients and possible risk factors of infection in this recently emerged endemic region. Further, this study tried to describe lymnaeid intermediate host snails involved in infection transmission morphologically and by molecular analysis. Analysis of the phylogenetic relationships between natural fasciolid populations was performed in humans, animals, and snail isolates by multilocus sequencing. Results indicated that asymptomatic fascioliasis should be screened in areas at risk of infection. Also, younger age groups and the presence of peri domestic animals were significantly associated with an increased risk of infection. This is the first molecular proof of the existence of *Radix auricularia* snails causing fascioliasis in Egypt. Results showed that Upper Egypt may be an area at risk for human fascioliasis that needs additional control measures.

## Introduction

Fascioliasis is an important food-borne zoonotic disease that poses a significant risk to humans. Fasciolosis is one of the neglected tropical diseases (NTDs) listed by the World Health Organization (WHO) [1]. In recent decades, the increase in the incidence of human fascioliasis and its outbreaks have introduced an emerging or re-emerging disease rather than a global zoonotic health problem [2]. Two major species, *Fasciola hepatica*, and *F*. *gigantica* are widely distributed and infect both domestic animals and humans [3]. The intermediate hosts of the two digenean species belonging to the family *Lymnaeidae* are different [4]. Infection with fascioliasis is caused by eating contaminated aquatic plants containing metacercariae or by drinking contaminated water [5]. *Fasciola* is primarily limited to the liver, resulting in hepatic lesions, fibrosis, and chronic inflammation of the bile ducts [6]. However, most infections are subclinical [5].

The diagnosis of infection in humans depends on a combination of different methods such as coprological examination to detect *Fasciola* eggs in the stool, indirect detection of anti-*Fasciola* antibodies or coproantigens by serological tests and imaging techniques such as abdominal ultrasonography (US) and/ or computed tomography (CT) [7, 8].

It was necessary to emphasize the connections between the lymnaeid vector species and *Fasciola*’s transmission pattern. In numerous regions, the distribution of *F*. *hepatica* among humans parallels its intermediate host snail, *Lymneaea* [5]. Several factors influence snail distribution in the environment, including the type of water collection, population dynamics, temperature thresholds, seasonality, and susceptibility to fascioliasis. Therefore, the identification of the snail vector responsible for disease transmission serves as an effective disease indicator for differentiating between potential disease scenarios and patterns and consequently, for determining the most effective control strategies [4]. Identification of lymnaeid snails is difficult due to their general uniformity and few morpho-anatomical characteristics, and sometimes even experts are unable to determine the precise species of the specimens [9]. Therefore, DNA sequencing data was recently used to help in determining the proper classification of snail specimens [10].

In temperate zones of Europe and Oceania, *F*. *hepatica* is the dominant species, and the only species in the Americas, whereas *F*. *gigantica* is widespread in Africa and Asia [11]. However, molecular studies throughout Africa, including Egypt, confirmed the existence of *F*. *hepatica* in multiple countries [12–14]. In addition, the geographical coexistence of both species has been extensively documented in many parts of Africa [14–17]. Several global studies have been conducted, including in Egypt, where both *Fasciola* spp. coexist and have documented the emergence of hybrids (or ‘intermediate forms’) of *Fasciola* [14, 18–21]. Consequently, it is difficult to distinguish *Fasciola* spp. based on morphological, clinical, coprological or immunological criteria, and the presence of hybrids further complicates the matter [22]. Recent molecular studies based on DNA analysis are now the most reliable method for identifying and characterizing the genetic makeup of morphologically similar fasciolids. The two digenean species and the intermediate form of *Fasciola* can be distinguished precisely by analyzing the ribosomal ITS1, ITS2, and 28S rRNA genes, as well as the mitochondrial (NADI) and (COXI) genes [14, 23–26].

Notably, ribosomal DNA (rDNA) has demonstrated its accuracy in the identification of *Fasciola* spp. due to the presence of conserved and variable regions and high copy numbers in these genes [27], whereas mitochondrial DNA (mtDNA) sequences exhibit higher mutation rates, lack of recombination, and maternal inheritance, therefore they are utilized as biomarkers for phylogenetic studies and genetic variability [28].

The phylogenetic classification of *Fasciola* spp. in Africa, including Egypt, particularly in livestock, has been extensively studied; however, there are very few studies describing the genetic structure of liver flukes that cause human fascioliasis. Human fascioliasis in Egypt was sporadic until the last three decades, when it became endemic, ranging from hypo- to hyperendemic [29], as observed in some villages in the Nile Delta [30]. However, the prevalence of human infections in Upper Egypt is likely underestimated, with only sporadic cases and few reports of outbreaks [31–33].

The present study aims to describe the risk factor analysis, sociodemographic and molecular characteristics of the fasciolid population in humans in a newly emerged endemic region in Assiut Governorate, Upper Egypt. Also, the identity of the lymnaeid intermediate host snails involved in this endemic area was examined by morphometric and molecular tools. We analyzed, for the first time in Upper Egypt, the phylogenetic relationships between natural fasciolid populations, in humans, animals, and snail vectors by multilocus sequencing.

## Materials and methods

### Ethics statement

Before contributing to the study, all participants gave their informed written consent. The study was approved by the Ethical Committee of institutional review board of the Faculty of Medicine, Assiut University.

### Study setting

This study was conducted on patients and their family members recruited from the outpatient clinic of Al-Rajhi Liver University Hospital, Assiut University. They were residents of the Manfalout neighborhood of Assiut Governorate, Upper Egypt, which experienced an outbreak of fascioliasis in 2018 and became endemic for the disease. Assiut Governorate, on the western bank of the Nile, is the study area for the participants who were recruited, and is located 350 kilometers (230 miles) south of Cairo, the capital, with geographic coordinates 27°19′N 30°58′E. It is primarily a rural community, where farming is the primary occupation of the residents and agricultural and pastoral activities are prevalent.

### Study population

Participants in the study were divided into two groups; the first group consisted of 74 patients recruited for the study during the period from August to October 2020 and exhibiting clinical symptoms suggestive of fascioliasis, including fever, jaundice, abdominal pain, and hepatomegaly. The second group consisted of asymptomatic close relatives of the patients (n = 65) who shared the same feeding habits and environmental conditions as the patients.

### Diagnosis of human fascioliasis

Each participant donated two blood samples (3 ml each) for the following laboratory screening tests.

- Complete blood count (CBC) with eosinophil % and absolute count.
- Indirect hemagglutination test (IHT) using Distomatose Fumouze kit (Laboratoires Fumouze Diagnostic, Levallois Perret, France) to detect anti-*Fasciola* antibody titer (a titer ≥ 1/320 was considered positive).

Individuals with high eosinophilia and a positive antibody titer underwent additional tests to assess their condition and confirm the diagnosis, including;

- Ultrasound of the abdomen (US) and/or CT.
- Endoscopic retrograde cholangiopancreatography (ERCP) was performed on patients diagnosed with obstructive jaundice by abdominal ultrasound and/or CT.
- *Fasciola* eggs were detected using a simple direct smear and formalin ether sedimentation technique [34] on stool samples. The presence of other intestinal parasites that may cross-react with fascioliasis or mask its symptoms was also investigated.

### Assessment of the risk factors for *Fasciola* infection

Consistent with similar previous studies, a detailed clinical history was recorded for all participants regarding sociodemographic characteristics and potential sources of infection based on the characteristics of the participants and the study locality [35, 36].

### Malacological study

#### 1. Snail collections and identification

Over the course of three months, a total of 600 lymnaeid snails (n = 200/month) were collected for the study. They were taken from public water reservoirs and irrigation canals in the Manfalout neighborhood of Assiut Governorate in Upper Egypt, where no permits were required. Local veterinarians reported foci of animal fascioliasis infection, which were used to select sampling locations. Lymnaeids were collected as described by Relf et al. [37] and were initially distinguished from other cohabiting freshwater snails based on the morphology of their shells, which featured dextral conical shells, triangular tentacles, and darkly pigmented eyes. After washing the snails onsite, they were immediately placed in moist, well-ventilated plastic containers for transport to the Parasitology laboratory. The collected snails were identified taxonomically using reference keys [38, 39].

#### 2. Detection and identification of fasciolid larval stages

The snails were washed with dechlorinated water. Each snail was then evaluated for *Fasciola* infections using two distinct techniques: *i*) testing for cercarial shedding [40]; *ii*) method of crushing [41]. All the observed stages were separated using a pipette for further research.

### Molecular study

#### 1. *Fasciola* and snail samples

Three adult flukes were collected from human patients with dilated common bile duct (CBD) and extracted by ERCP. Two flukes were collected from cattle’s infected livers from the same locality. Two pooled samples contained head–foot tissue parts of infected lymnaeid snails and two pooled samples contained head–foot tissue parts of noninfected lymnaeid snails that served as controls for molecular sequencing. Before DNA extraction, the lateral margins of the posterior portion of each fluke, excluding the uterus and ootype, were used. The collected snails were processed according to Kulsantiwong et al. for DNA extraction [42]. The manufacturer’s instructions for the DNeasy Blood & Tissue Kit (Qiagen, USA) were followed for DNA extraction, and an Epoch Spectrophotometer was used to estimate DNA concentration.

#### 2. PCR amplification and sequencing

For the characterization of fasciolid flukes and lymnaeid snails, PCR amplification of the rDNA ITS1 and mtDNA NADI and COXI genes was performed with the same reaction volumes and cycling conditions as previously reported by Itagaki et al. [20]. Ultraclean DNA purification kit (Qiagen, USA) was used to purify PCR products according to the manufacturer’s instructions. At the SolGent sequencing facility, sequencing reactions were performed with a BigDye Terminator v3.1 Cycle Sequencing Kit (Applied Biosystems, Foster City, CA, United States) according to the manufacturer’s instructions (SolGent Co., Ltd, South Korea). Each marker’s accuracy was confirmed through bidirectional sequencing. An ABI 3130 Genetic Analyzer was used to analyze the sequencing data (Applied Biosystems, Foster City, CA, United States).

#### 3. Sequence analysis

GenBank Blast https://blast.ncbi.nlm.nih.gov/Blast.cgi) identified the similarity between the two sequences. The sequences were aligned with each other and reference sequences using **http://www.megasoftware.net** with the default settings of MUSCLE in MEGA X.0.[43]. The COX1 and NAD1 mitochondrial gene sequences were translated into amino acids to detect the possibility of pseudogene amplification. The evolutionary relationship between ribosomal ITS1 and mitochondrial genes was determined by constructing phylogenetic trees using maximum likelihood (ML) and the neighbor-joining distance method with a confidence level of 1000 bootstrap replicates. As the outgroup, the nucleotide sequences of *Fascioloides magna* (EF534991, EF535001, and GU599871) were used to root phylogenetic trees.

#### 4. Haplotype analysis

DnaSP 5.1 software [44] was utilized to calculate the diversity indices (Haplotype (Hd) and nucleotide (π) diversities) between the present isolates of *F*. *hepatica* and *F*. *gigantica*, and the reference flukes.

### Statistical analysis

Participants’ demographic data and relevant risk factors were analyzed by IBM-SPSS 24.0 (IBM-SPSS Inc., Chicago, IL, USA). Means, standard deviations, median, interquartile range (IQR), frequency, and percentages were calculated for descriptive data. Significance tests: The Chi-square test was used to compare group frequencies. Post-hoc tests were calculated using Bonferroni corrections for pairwise comparisons between the study groups. One-way ANOVA was used to test for differences in means of data with normal distribution, while the Kruskal–Wallis non- parametric test was used to compare differences in the median of data without normal distribution. Subsequently, the association between variables was examined using multivariate logistic regression analysis. A *p*-value was considered significant when <0.05.

## Results

### Demographics and risk factor analysis

This study included a total of 139 participants (68 male and 71 female). Seventy-four had acute or chronic fascioliasis, while the remaining 65 were asymptomatic relatives. The participants’ demographic characteristics and disease-related information are summarized in **Table 1**.

**Table 1:** Baseline characteristics and disease-related data of the studied population showing the relationship between determinants of infection and fascioliasis.

	Total (n = 139)	Cases (I) (n = 74)	Positive Relatives (II) (n = 20)	Negative Relatives (III) (n = 45)	*P*-value
Age/year (mean ± SD)	29.52 ± 1.2	26.82 ± 1.3	27.60 ± 1.4	34.80 ± 1.7	= 0.028*
*P*-value**		**I vs. II = 0.847**	**II vs. III = 0.045**	**I vs. III = 0.009**	
Sex (n, %)					= 0.211***
• Male	68 (48.9%)	32 (43.2%)	13 (65%)	23 (51.1%)	
• Female	71 (51.1%)	42 (56.8%)	7 (35%)	22 (48.9%)	
Occupation (n,%)					= 0.011***
• Housewife	36 (25.9%)	25 (33.8%)	3 (5%)	8 (17.8%)	
• Student	51 (36.7%)	32 (43.2%)	7 (35%)	12 (26.7%)	
• Farmer	27 (19.4%)	8 (10.8%)	5 (25%)	14 (31.1%)	
• Skilled	10 (7.2%)	0 (0%)	3 (15%)	7 (15.6%)	
• Professional	15 (10.8%)	9 (12.2%)	2 (10%)	4 (8.9%)	
Education (n,%)					= 0.045***
• Illiterate	36 (25.9%)	26 (35.1%)	2 (10%)	8 (17.8%)	
• Basic	75 (54%)	37 (50%)	14 (70%)	24 (53.3%)	
• University	28 (20.1%)	11 (14.9%)	4 (20%)	13 (28.9%)	
Residence (n,%)					= 0.009***
• Rural	89 (64%)	52 (70.3%)	16 (80%)	21 (46.7%)	
• Urban	50 (36%)	22 (29.7%)	4 (20%)	24 (53.3%)	
Piped Water supplies (n,%)	107 (77%)	58 (78.4%)	12 (60%)	37 (82.2%)	= 0.133***
Eating raw vegetables (n,%)	112 (80.6%)	63 (85.1%)	15 (75%)	34 (75.6%)	= 0.182***
Peridomestic animals (n,%)	97 (69.8%)	60 (81.1%)	16 (80%)	21 (46.7%)	< 0.001***
Eosinophils percent (Med ± IQR)	14 (1–72)	35 (18)	8 (3)	3 (1)	< 0.001$
Cases with high eosinophilia (n,%)	89 (64%)	67 (90.5%)	19 (29.2%)	3 (4.6%)	< 0.001***
*P*-value**		**I vs. II < 0.001**	II vs. III = 0.051	**I vs. III < 0.001**	
Hepatic focal lesions (n,%)	34 (24.5%)	26 (35.1%)	8 (40%)	0 (0%)	< 0.001***

* ANOVA test was used to compare the mean difference between groups

**Post-hoc test was used for pairwise comparison with Bonferroni correction

***Chi-square test was used to compare frequency between groups

^$^Kruskal Wallis test was used to compare the median difference between groups

Med ± IQR: Median ± Interquartile range.

High mean eosinophilia 21.92 ± 17.3, positive anti-*Fasciola* antibody titer (936.1 ± 387.2), and hepatic focal lesions (HFLs) in US confirmed the diagnosis (24.5%). Moreover, in 9.46% of patients, the stool examination was positive.

In order to detect possible asymptomatic *Fasciola* infection, the relatives of patients were screened for anti-*Fasciola* antibodies and eosinophilia in their sera using IHT and CBC. Anti-*Fasciola* antibodies were detected in 20/65 (30.8%) asymptomatic relatives and elevated eosinophilia (>6%) was detected in 19/65 (29.2%). However, the US examination revealed FHLs in 8 of these relatives. The analysis of variance revealed that the prevalence of fascioliasis was significantly higher among young students, particularly those with primary education. Those living in rural areas had higher prevalence rates. Significantly more peridomestic animals were found near patients and their positive relatives. Other factors, including the consumption of raw vegetables such as watercress, coriander, and lettuce, and the use of piped water supplies, did not differ significantly.

The relationship between *Fasciola* infection and the sociodemographic characteristics of the participants is depicted in **Table 2**. The risk of infection was significantly related to the age of the participants (younger populations have a higher risk of infection) (*P* = 0.007) and the proximity of farm animals to human dwellings (*P*< 0.001). The odds of association between animal breedings and the risk of *Fasciola* was 5.89 (CI 95%: 2.196–15.827), indicating that animals play a significant role in disease transmission in this area.

**Table 2:** Logistic regression of the risk factors for fascioliasis infection.

Item	*P* value	Odds ratio	Confidence Interval 95% Lower level Upper level	
**Age**	0.007*	0.961	0.934	0.989
**Sex**	0.539	1.4	0.476	4.134
**Occupation**	0.293	0.798	0.524	1.215
**Education**	0.723	0.890	0.467	1.697
**Residence**	0.198	0.545	0.216	1.372
**Eating raw vegetables**	0.715	0.821	0.284	2.371
**Animals in predomestics**	0.000*	5.896	2.196	15.827
**Water treatment**	0.840	0.894	0.301	2.656

*Significant P- value.

### Morphological identification of lymnaeid snails and fasciolid developmental stages

**Fig 1:**
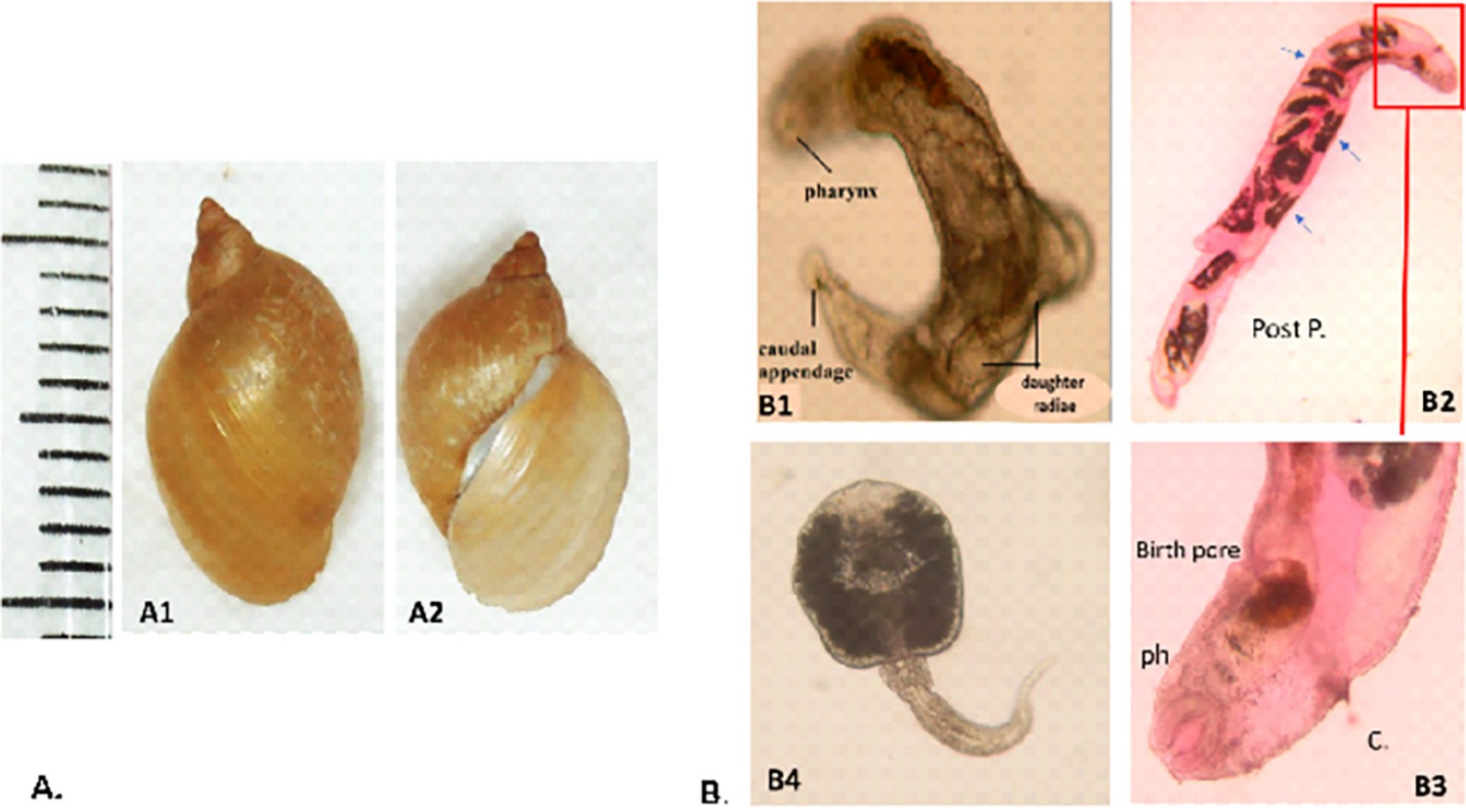
**A. Shell of *R*. *Auricularia* collected snails from Assiut, Upper Egypt. A1**: Dorsal view; **A2:** Ventral view. **B. Light micrograph of *Fasciola* larval stages collected from *R*. *auricularia* snails. B1:** Mother redia showing the pharynx (ph.), collar (c.), and a caudal papilliform process ×40. **B2:** Daughter redia showing the developing cercariae (arrowheads) and a posterior process (post.p.) ×40. **B3**: Magnification of anterior portion showing the pharynx (ph.), collar (c.), and birth pore x100. **B4**: *Fasciola* spp. Cercaria ×100.

Six hundred lymnaeid snails from Manfalout locality, Assiut, Upper Egypt, were collected, identified, and examined for the presence of *Fasciola* larvae. Lymnaeid snails were distinguished morphologically as *Radix auricularia* based on their elongated, thin, nonoperculated, somewhat fragile shell with a large dextral shell opening and sharp apex. There were four whorls, with the final one having a leftward convex angle. The surface of the shell appeared smooth, thin, and brown with distinct ribs. The aperture was large and shaped like an ear, with a curved columella in the center. The outer lip’s lower portion was rounded (Fig 1A).

Twenty-nine percentage of the collected snails were infected with various *Fasciola* developmental stages, including sporocysts, rediae, and cercariae. Fig 1B depicts the microscopic features of these stages.

### Molecular analysis of *Fasciola* adult based on ribosomal ITS-1

The nucleotide sequences of *Fasciola* specimens have been deposited in GenBank under accession numbers as shown in Table 3. The ITS-1 sequences included the small ribosomal subunit RNA gene, a partial sequence of the 18S, ITS1, and 5.8S rRNA genes, and a total of 591–617 base pairs.

**Table 3:** Comparison of the ITS-1 sequences of *F*. *hepatica* and *F*. *gigantica* of the present study with those from different hosts and geographical localities in GenBank.

Species	Locality Accession no	Host	Nucleotide sites of ITS1 region						
			79	103	207	297	391	469	489
***F*. *hepatica* FH4 ITS1**	Assiut (MW248564)	Human	T	T	C	A	C	T	C
***F*. *hepatica* FH3 ITS1**	Assiut (MW248571)	Human	T	T	C	A	C	T	C
***F*. *hepatica* FH5 ITS1**	Assiut (MW248568)	Human	T	T	C	A	C	T	C
***F*. *gigantica* ITS1**	Assiut (MW219536)	Cattle	C	C	T	T	T	A	T
***F*. *gigantica* ITS1**	Assiut (MW219535)	Cattle	C	T	T	T	T	A	T
***F*. *hepatica* ITS1**	Egypt (LC076196)	Sheep	T	T	C	A	C	T	C
***F*. *hepatica* ITS1**	Iran (GQ925431)	Human	T	T	C	A	C	T	C
***F*. *hepatica* ITS1**	India (KX198629)	Sheep	T	T	C	A	C	T	C
***F*. *hepatica* ITS1**	Japan (AB514847)	Cattle	T	T	C	A	C	T	C
***F*. *gigantica* ITS1**	Viet Nam (MN970008)	N.A	T	T	C	A	C	T	C
***F*. *hepatica* ITS1**	Australia (MF678648)	Sheep	T	T	C	A	C	T	C
***F*. *hepatica* isolate ABC-02**	France (JF294999)	N.A	T	T	C	A	C	T	C
***F*. *hepatica* isolate Bca2**	Argentina (MG201869)	Buffalo	T	T	C	A	C	T	C
***F*. *hepatica* ITS1 Egypt**	Egypt (KP099942)	N.A	T	T	C	A	C	T	C
***F*. *hepatica* ITS1**	Tunisia (JF423939)	Equine	-	-	C	A	C	T	C
***F*. *gigantica* ITS1**	Chad (MK321606)	Sheep	-	-	T	T	T	A	T
***F*. *gigantica* ITS1**	Kenya (KP760871)	Connochaetes taurinus	-	-	-	T	T	A	T
***F*. *gigantica* ITS1**	Chad (MK321641)	Cattle	-	-	-	T	T	A	T
***F*. *gigantica* ITS1**	Zimbabwe(MW046874)	*Radix natalensis*	-	-	-	T	T	A	T
***F*. *hepatica* ITS1**	Egypt (KP215281)	Human	-	-	-	A	C	T	C

N.A = not available

Alignment analysis using online NCBI Blast confirmed the conservation of the 18S and 5.8S rDNA within *Fasciola* sp. and revealed that ITS-1 sequences of human samples (MW248564, MW248568, and MW248573) were 100% identical to those of *F*. *hepatica* which were previously published, while those of animal samples with accession No. (MW219535 and MW219536) were 97–100% identical to those of *F*. *gigantica* reference sequences from different countries. The presence of seven polymorphic sites indicative of the occurrence of both *F*. *hepatica* and *F*. *gigantica* inferred from multiple alignments of ITS-1 sequences with *Fasciola* spp. sequences available in GenBank to infer the annotation. No nucleotide deletions were observed between the two *Fasciola* species. The primary difference between *F*. *hepatica* and *F*. *gigantica* was a single-base substitution: A > T at the nucleotide site of 469. T > A at the nucleotide site of 297, C > T at positions 79 and 103, and T > C at the sites 207, 391, and 489 (Table 3).

**Fig 2:**
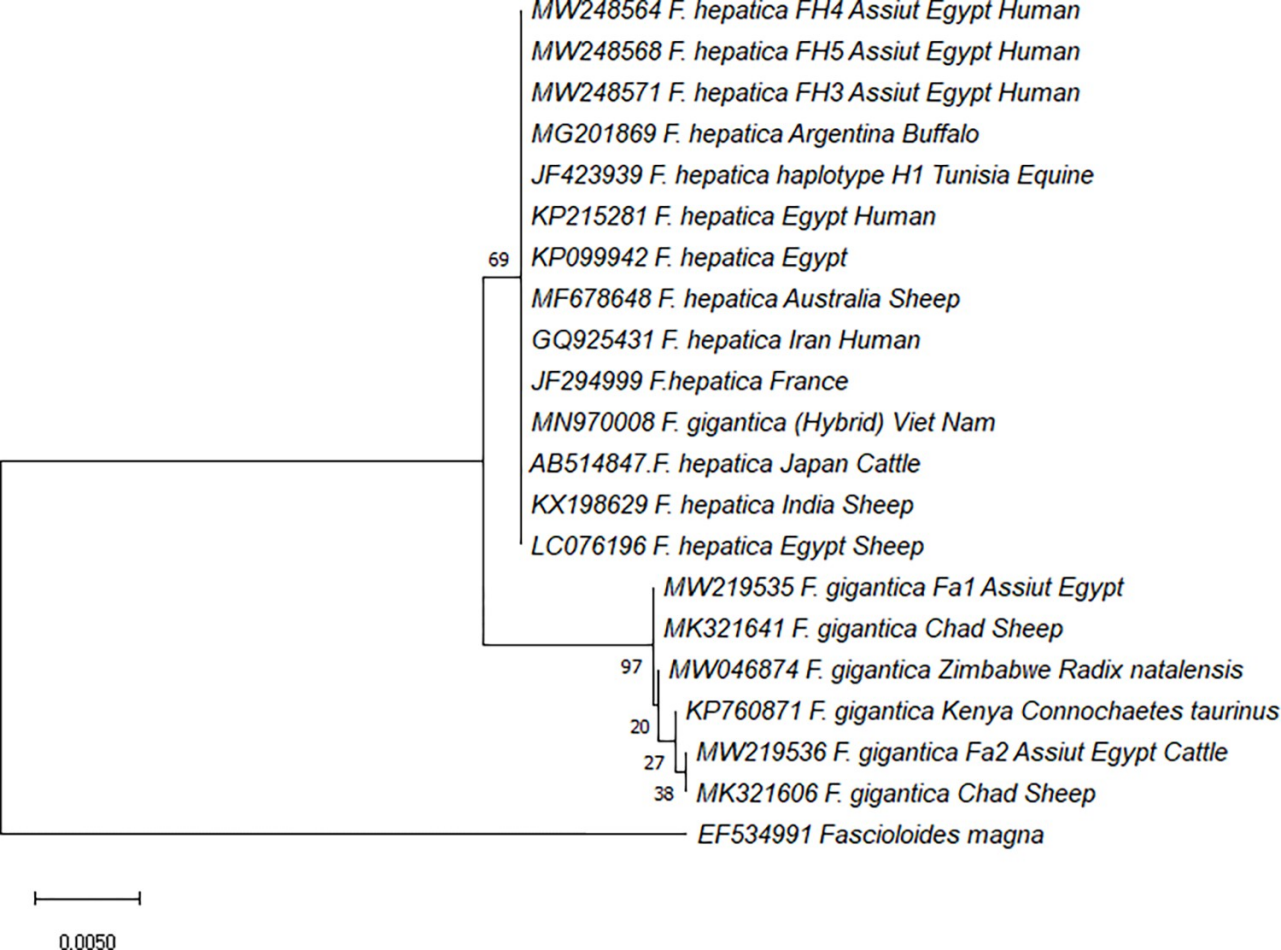
The phylogenetic relationships based on the ribosomal ITS-1 analysis: Including specimens collected from humans and cattles in Upper Egypt and other representative isolates in the GenBank database from different localities. Maximum likelihood method and Kimura 2-parameter model were used with the percentage of bootstrap confidence level (1000 replicates) This analysis involved 21 nucleotide sequences with a total of 420 positions in the final dataset.

*F*. *hepatica* and *F*. *gigantica* isolates from Assiut, Upper Egypt were compared to the most closely related reference isolates using the ML method to generate a phylogenetic tree. The ITS-1 phylogenetic tree revealed that *Fasciola* flukes were distributed into two main clades of pure *F*. *hepatica* and *F*. *gigantica*, except for a hybrid isolate of *F*. *gigantica* from Vietnam (MN970008), which was merged into the *F*. *hepatica* clade. It was demonstrated that the main clade of *F*. *hepatica* included isolates from humans of the present study and those obtained from various animals from different countries with a bootstrap confidence level of 69%. The *F*. *gigantica* clade consisted of two main clusters that were further divided into terminal subclusters involving *F*. *gigantica* isolates removed from cattle in this study (MW219535 and MW219536) with the same species retrieved from different hosts (97% bootstrap value) (Fig 2). There was no geographic or host segregation in both *F*. *hepatica* and *F*. *gigantica* sequences. The neighbor-joining tree (NJ) yielded similar results, confirming the same topology structure of the phylogenetic trees with high bootstrap confidence levels (S1 Fig).

### Molecular analysis of lymnaeid snails

The ITS-1 molecular analyses of *Radix* spp. resulted in DNA fragments of approximately 720 bp in length, which were deposited in GenBank under accession no (MW248565, MW248569, MW248575, and MW248576). Alignment analysis using online NCBI Blast showed that the study specimens shared the highest similarities with *R*. *auricularia* from other countries (89.36% with HG932017, JF922878 from Germany and Spain, respectively, 88.63% with JX193589 from Netherlands, and 83.96% identity with *R*. *auricularia* MN194260 from India). It was noted that only one isolate of *R*. *natalensis* (HQ283257) from France shared 87.85% identity with our samples.The obtained sequences contained a portion of the small ribosomal subunit, the entire ITS-1 sequence, and a portion of the 5.8S sequence.

**Fig 3:**
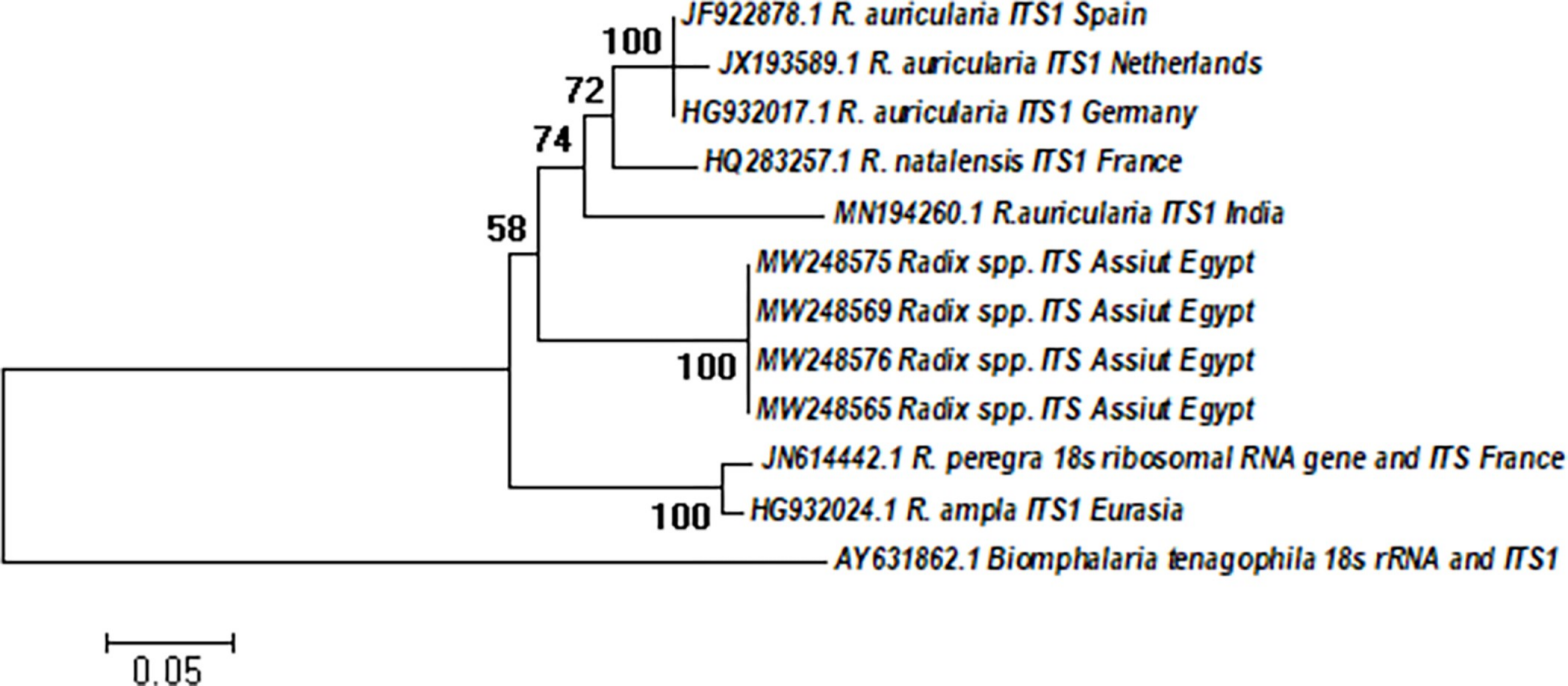
The phylogenetic relationships of *Radix* spp. based on ITS- 1 region: For lymnaeid specimens collected in this study and other representative isolates in the GenBank database from different localities. Phylogenetic trees were constructed using MEGA X.0 with bootstrap values of 1000 replicates set for NJ method and Tamura-Nei model.

The sequences are grouped into two primary clusters in the phylogenetic tree based on NJ analysis (Fig 3). The first cluster included the GenBank reference sequences of *R*. *auricularia*, our samples, and *R*. *natalensis* with a bootstrap confidence level of 58%. While those of *R*. *peregra* and *R*. *ampla* sequences gathered into a separate cluster with a bootstrap confidence level of 100%. In the same vein, the ML method analysis was identical to the NJ tree, which revealed two significant clusters (S2 Fig).

### Mitochondrial gene analysis

After trimming the sequences, amplification of the COXI mitochondrial gene produced DNA products (421–430 bp) that were specific to *F*. *gigantica* and *F*. *hepatica*, respectively. However, NADI sequences generated approximately (578–581 bp). At Blastn, the studied samples exhibited a high degree of similarity, ranging from 97% to 100%, with previously published sequences of *F*. *gigantica* and *F*. *hepatica* recovered from various localities and stored in the GenBank database. The alignment of COXI mtDNA nucleotide sequences with reference sequences generated 7 haplotypes (4 haplotypes named FgCOI-H for *F*. *gigantica* and 3 haplotypes named FhCOI-H for *F*. *hepatica*). For *F*. *hepatica* and F. *gigantica*, the mitochondrial NADI gene generated 9 haplotypes (3 FhNDI-H and 6 FgNDI-H, respectively). Tables 4 and 5 illustrate the haplotype numbers, sample accession numbers, and geographic distribution.

**Table 4:** Comparison of the NADI sequences of *Fasciola* spp. from Upper Egypt with those from different geographical localities.

Species	Country	Host	Haplotypes	Accession number	Reference
*F*. *hepatica*	Iran Poland Egypt Armenia	Human Sheep Sheep Cattle	FhNDI-H1	GQ175362 KR422395 LC076257 MG972401	[45] [46] [47] [48]
	Egypt* Egypt* Egypt* Egypt* Egypt*	Human Human Human Snail Snail	FhNDI-H1	MW218441 MW218442 MW218443 MW218444 MW218445	— - - -
	Spain	Sheep	FhNDI-H2	KF111680	[49]
	Brazil	Cattle	FhNDI-H3	MK838728	[50]
*F*. *gigantica*	Iran	Cattle	FgNDI-H1	KX021288	[45]
	Egypt* Egypt	Cattle Sheep	FgNDI-H2	MW218440 LC076211	- [47]
	Nigeria	Bos_indicus	FgNDI-H3	LC142768	[51]
	Ghana	Cattle	FgNDI-H4	MF490247	[52]
	Maghreb	Sheep	FgNDI-H5	MN913874	[53]
	Egypt*	Cattle	FgNDI-H6	MW209692	-

* = Sequences of the present study

**Table 5:** Comparison of the COXI sequences of *Fasciola* spp. from Upper Egypt with those from different geographical localities.

Species	Country	Host	Haplotypes	Accession number	Reference
***F*. *hepatica***	Egypt*	Human Human Snail Snail	FhCOI-H1	MW217462 MW217468 MW217463 MW217465	-
	Egypt	Buffalo	FhCOI-H1	AB553828	[14]
	Iran Poland Iran Ecuador	Sheep Bison_bonasus Goat Cattle	FhCOI-H2	MN527599 KR422385 MK447990 LC273058	-
	Egypt*	human	FhCOI-H3	MW217461	-
***F*. *gigantica***	Egypt* Egypt*	Cattle Cattle	FgCOI-H1	MW217470 MW217466	- -
	Egypt Iran Mauritania	Buffalo Cattle Sheep	FgCOI-H1	AB553784 KX021275 HQ857101	[14] [54] [55]
	India	Bos_frontalis	FgCOI-H2	KX656877	[56]
	Viet Nam	Cattle	FgCOI-H3	MF287791	-
	Turkey	Buffalo	FgCOI-H4	KY613945	[57]

* = Sequences of the present study

**Fig 4:**
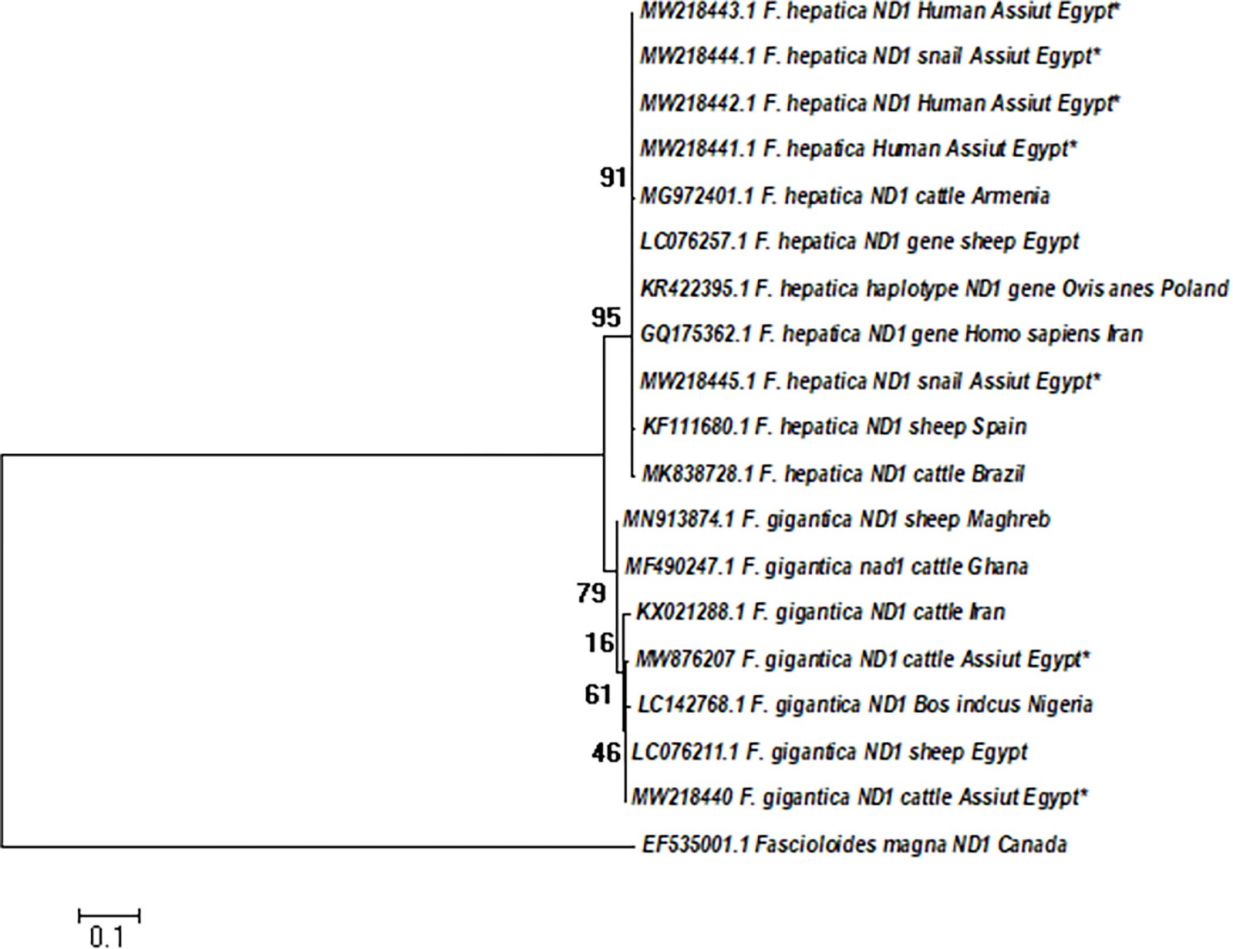
ML tree based on partial sequence of NADI gene constructed by MEGA X.0. [43]: showing phylogenetic relationships of Egyptian *Fasciola* isolates obtained in this study (marked with Asterix*) compared to reference NAD1 sequences in the GenBank database. The percentage of bootstrap replicates (1000 replicates) is shown next to the branches The evolutionary history was inferred by using the Maximum Likelihood method and Tamura-Nei model.

Furthermore, the evolutionary relationship between the studied isolates and reference strains from other countries based on mitochondrial NADI gene analysis was determined using ML and NJ phylogenetic trees (Figs 4 and S3) in MEGA X.0, which revealed nearly identical evolutionary tree structures. ML analysis showed that *Fasciola* isolates of the present study from humans, animals, and snails were grouped into two main clades of *F*. *hepatica* and *F*. *gigantica*. The sequences belonging to the *F*. *hepatica* clade showed only 5 DNA variable sites segregated on 3 haplotypes where all Egyptian *F*. *hepatica* isolates clustered into a distinct haplotype (designated as Fh-NDIH1) with a high bootstrap value > 90%. While those of *F*. *gigantica* showed more genetic diversity at this marker with 18 DNA variable sites producing 6 haplotypes. The alignment clustered one *F*. *gigantica* isolate of the present study with an Egyptian isolate (LC076211, [47]) into a distinct haplotype (FgNDI-H2) while the other isolate generated a separate FgNDI-H6 Haplotype (Table 4).

**Fig 5:**
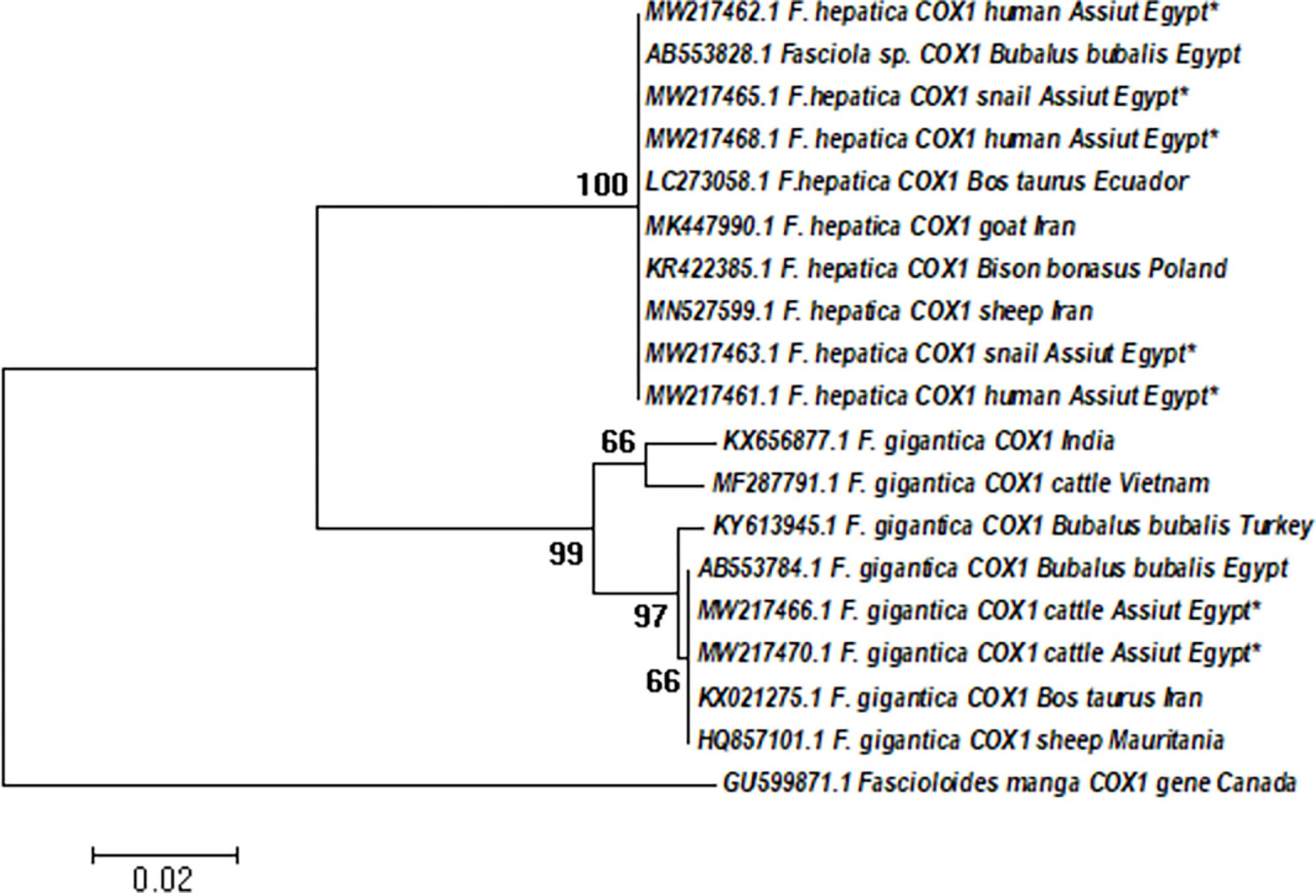
Phylogenetic tree based on partial sequence of COXI gene constructed using ML method and Tamura-Nei model using MEGA X: the Egyptian *Fasciola* isolates of the study were marked with Asterix* compared to reference COXI sequences in the GenBank database. The bootstrap confidence level inferred from 1000 replicates is taken next to the branches.

In accordance with the findings of NADI gene, the obtained partial sequences of the COXI gene revealed nearly the same evolutionary history of *F*. *hepatica* and *F*. *gigantica* groups (Figs 5 and S4). In addition, *F*. *hepatica* isolates of the present study revealed low genetic diversity with only 2 segregating sites indicating 3 haplotypes that include *F*. *hepatica* human isolate (MW217461) in a separate haplotype (FhCOI-H3) while the remaining of *F*. *hepatica* isolates were clustered with an Egyptian isolate (AB553828), reported by Amer et al. [14] as Fh-COIH1 haplotype.

Regarding to *F*. *gigantica* group, 4 haplotypes demonstrating 13 DNA segregating sites were recorded. Phylogenetic analysis showed that Egyptian isolates of the *F*. *gigantica* group clustered (bootstrap value 66%) with other sequences from Egypt, Iran, and Mauritania forming a distinct haplotype (FgCOI-H1) (Table 5)

### Haplotype analysis

The diversity indices of mitochondrial genes including the haplotype diversity (Hd), nucleotide diversity (Pi), and the number of segregating sites (S) were calculated in both *F*. *hepatica* and *F*. *gigantica* groups to compare our isolates with reference sequences. There was low genetic diversity in *F*. *hepatica* isolates compared with reference sequences of both markers. However, the diversity was higher in *F*. *gigantica*, group, especially for the NADI gene (Table 6).

**Table 6:** Diversity indices of mitochondrial DNA subunits in relation to *F*. *gigantica* and *F*. *hepatica* groups.

		No of haplotypes	Haplotype diversity (Hd)	Variance of haplotype diversity	Nucleotide diversity (Pi)	Number of segregating sites (S)
**NAD1**	*F*. *hepatica*	3	0.345	0.02967±0.172	0.0020	5
	*F*. *gigantica*	6	0.952	0.00912±0.096	0.0134	18
**COX1**	*F*. *hepatica*	3	0.644	0.01025±0.10	0.00199	2
	*F*. *gigantica*	4	0.643	0.03390±0.184	0.01090	13

## Discussion

Fascioliasis remains one of the most significant NTDs, particularly in endemic nations like Egypt [47]. In Egypt, fasciolosis is endemic in the Nile Delta (Lower Egypt) [31, 58] and causes serious human and animal health problems [47]. However, the situation in Upper Egypt is poorly understood, and precise infection estimates are lacking. Human fascioliasis reports from Upper Egypt were infrequent until 2015, and the incidence of human cases is on the rise in Assiut Governorate, Upper Egypt [31, 32], with a recent outbreak in 2018 [33]. Determining the snail infection rate, the epidemiological criteria, the risk factors, and a prior genetic characterization have become fundamental requirements for preventing further spread or outbreak of this devastating disease.

In this newly emerged endemic region in Upper Egypt, the sociodemographic characteristics of the studied sample likely explain the emergence and describe the distribution of fascioliasis and the risk factors for human infection. Younger age groups had a higher infection rate, representing a significant risk factor, according to the current findings. A significant finding of this study is the high prevalence of fascioliasis infection among elementary school children. Previous studies conducted in Egypt and other hyperendemic regions reported this observation [29, 36, 59–62]. They might become infected by eating uncooked contaminated plants while helping their parents in farming or by drinking contaminated water while playing in drainage canals [63, 64]. This observation has been reported in other studies conducted in several South American and Middle Eastern countries [32, 65].

The results regarding the distribution of fascioliasis by gender are frequently inconsistent [66]. The present study showed no correlation between gender and infection; both males and females were infected. Several European regions reported a similar finding [3]. In other hyperendemic regions, however, females were considered the commonly infected group [29, 60].

In addition, residents of rural areas with nearby domestic animals such as cattle, sheep, etc., had a higher infection rate than those living in urban areas with no peridomestic animals. In similar hyperendemic regions of Egypt, researchers such as Curtale et al. reported that the proximity of farm animals to farmers’ dwellings posed a risk [67–69]. A recent study revealed that contact with domestic animals and the presence of animal dwellings in the households of Egyptian farmers posed a risk of *Fasciola* transmission to consumable vegetables, utensils, and drinking water supplies, and subsequently to the farmers’ wives and children [3]. This can explain the significant association between peridomestic animal presence and infection risk in the present study.

This study emphasizes the importance of eosinophilic count and anti-*Fasciola* antibody screening in the families of *Fasciola*-infected patients. As documented previously, a single case of fascioliasis may indicate a familial or local outbreak [70]. Members of the same family are at risk of contracting the infection because they share the same eating habits or infection sources, such as contaminated food or water [62]. These patients may continue to be asymptomatic; consequently, eosinophilic count and serological screening of their sera are necessary to prevent loss of diagnosis in asymptomatic cases. Herein, 30.8% (20/65) of the asymptomatic relatives screened were positive for anti-*Fasciola* antibodies with elevated eosinophilia (>6%). In addition, eight of them (8/20) exhibited radiological evidence of fascioliasis (HFLs) on ultrasound and/or CT. Similar observations were previously reported in Egypt [71], Spain [72], Eastern Anatolia [70], and Peru [73]. These findings validate the utility of serological-based tests and radiological methods as dependable diagnostic tools in community-based surveys for fascioliasis, particularly in the early stages of infection when diagnostic eggs are absent from the stool, and clinical abnormalities have not yet developed [70].

Similar to other vector-borne parasitic diseases, fascioliasis is transmitted via freshwater bodies and *lymnaeid* snails, which serve as *Fasciola*’s major global intermediate hosts [39, 74]. Consequently, it is essential to regularly update the information, faunal list, and snail distribution, especially as new endemic foci emerge [4, 75]. At present, Egypt is a home to five species of lymnaeids, including *Galba truncatula*, *Galba schirazensis*, *Lymnaea stagnalis*, *Pseudosuccinea columella*, and *Radix natalensis* [75, 76]. *Radix natalensis* was the chief vector for *Fasciola* in Qena, Upper Egypt [77], and is the specific vector for *F*. *gigantica* in Africa [9].

The collected snails in this study were identified as *R*. *auricularia* based on the morphological characteristics of their shells. The typical form of *R*. *auricularia* is distinguished by its ear-shaped shell and greatly expanded body whorl [78]. However, the proportions and shapes of its shells vary widely owing to the plasticity of shell morphology affected by environmental pressures and ontogeny [79]. On the other hand, most of *Radix* spp. expressed a relative similarity in their morphological criteria in response to adaptations to shared environments. Therefore, accurate identification between species is extremely difficult [80]. Thus, molecular approaches to speciation are the most reliable [81]. The sequencing of *lymnaeid* DNA began at the end of the 19th century, and its advanced application revealed the problems in the species differentiation posed by conventional phenotypic malacological studies. Therefore, definitive characterization is only achievable through marker sequencing [10].

Molecular analyses of *Radix* spp. confirmed the presence of *R*. *auricularia* in the study area. Our sequences were grouped with *R*. *auricularia* reference sequences from different countries through sequencing and phylogenetic analysis. Notably, Egyptian *Radix* isolates have a separate position on the phylogenetic trees (Fig 3), which is supported by a high bootstrap value reflecting the prominent nucleotide differences from reference sequences. Such variations may be attributed to the genetic heterogeneity of *Radix* spp. in this newly emerged area in Egypt which may indicate the occurrence of unique and uniform evolutionary events with the introduction of this snail into novel geographic localities. The presence of geographically discrete clusters of *R*. *auricularia* isolates has been previously reported [80, 82].

In addition, a European isolate of *R*. *natalensis* (HQ283257) was clustered in close relatedness with our isolates in the same *R*. *auricularia* group. Such interesting finding may indicate a potential Old-World origin along with genetic overlap with *R*. *natalensis* populations widely distributed in Egypt. In consistent with our results, Lawton *et al*. reported that UK and Eurasian isolates of *R*. *Auricularia* were clustered with the African *R*. *natalensis* and the Asian *R*. *rubiginosa* owing to the wide distribution of this species allover continents [80]. Furthermore, Bargues *et al*. indicated that changes in the phylogenetic topology and the occurrence of interrelatedness between clades could be confounded by the type of genetic marker used for analysis [10]. Therefore, some researchers illustrated the usefulness of mitochondrial genes such as COXI gene over nuclear genes in differentiating lymnaeid snails and in providing a comprehensive biogeographical perspective [80, 82].

To the best of our knowledge, no molecular studies on *R*. *auricularia* have been conducted in Egypt to date. This is presumed to be the first set of data on the molecular identification and phylogenetic analysis of this snail species in Egypt based on rDNA analysis methods. Due to the lack of recent records, it is believed that this medically significant snail species has become extinct in Egypt [75, 83]. Notably, *R*. *auricularia* was last reported in the country in 1968 [84]. The repopulation and spread of this snail species in the area under study may be the result of climate change and inadequate control measures [32].

Our findings have shown that the current Egyptian snail isolate serves efficiently as an intermediate host for *Fasciola*, as 29% of the collected snails were found to be naturally infected with different larval stages of *Fasciola* **(**Fig 1**)**. The infection by *F*. *hepatica* haplotypes was confirmed through a molecular examination of snail tissues using mtDNA markers NADI and COXI. This highlights the role of the identified snails in the emergence and distribution of fascioliasis infection in humans and animals, which parallels its distribution in snail hosts [85]. This association was explained by the spatial distribution of snail hosts and their habitats in relation to *Fasciola* prevalence and intensity of infection [86]. *R*. *auricularia* likely played a significant role in the introduction, expansion, and genetic diversity of the *Fasciola* population in the study area.

Modern molecular techniques have been widely applied to the characterization of *Fasciola* isolates exhibiting overlapping distribution in several African and Asian nations [6, 47, 87]. DNA sequencing provides clear differentiation between the two *Fasciola* spp. and the intermediate forms (hybrid and introgressed) based on multiple markers, including ribosomal and mitochondrial markers [25, 88]. To examine the population genetic structure of *Fasciola* spp. collected from *Fasciola*-positive patients, animals, and snail hosts, molecular analysis was conducted using genomic ITS-1 and mitochondrial (NADI and COXI) gene markers that have been demonstrated to be reliable for the identification of *Fasciola* species [21].

Sequence analysis of the ITS-1 gene confirmed the co-occurrence of *F*. *hepatica* and *F*. *gigantica* in Upper Egypt, corroborating a previous finding [89, 90]. Through molecular characterization, *F*. *hepatica* has been identified in human and snail isolates while *F*. *gigantica* has been detected in bovine isolates. Furthermore, *F*. *hepatica* infection in human samples was previously identified genetically using rDNA ITS markers in Upper Egypt [91] and Iran [92]. These reports demonstrate the remarkable adaptability of *F*. *hepatica* flukes to human hosts. In contrast, a previous study conducted in Vietnam recovered a ‘pure’ *F*. *gigantica* isolate from nine patients using nuclear ribosomal ITS1 and ITS2 markers [19].

This study established that *F*. *hepatica* was the dominant species infecting humans and snails while *F*. *gigantica* is the isolated strain from cattle in the same endemic area [90]. These findings contradict the recent findings of Omar et al., who reported that *F*. *hepatica* was predominant in cattle [90]. Previous research conducted in Egypt revealed that *F*. *gigantica* is the predominant species found in cattle and buffaloes [14]. Earlier reports from elsewhere in the world indicated that *Fasciola* spp. exhibited a wide variation in relation to the type of host. *F*. *hepatica* was the predominant species in cattle in certain African nations, such as Algeria and South Africa [17, 93], whereas *F*. *gigantica* is more prevalent in Zimbabwe and certain Asian nations, such as Thailand [94] and Vietnam [19]. This variation in *Fasciola* species distribution may be attributable to environmental, snail susceptibility, and other host-related factors. Due to the limited number of samples collected from animal hosts and the lack of host variability, it was not possible to determine the predominant *Fasciola* species inhabiting different animal hosts in the studied area.

mtDNA is maternally inherited and traces the maternal lineage of single species or hybrid forms. This is due to the high ratio of copies and rapid mutation rate, which produce sequence differences among closely related species or subspecies [95]. As revealed by the molecular analysis of the mt COXI and NADI genes, *Fasciola* isolates from the current study shared a high degree of similarity, up to 100%, with previously published sequences of *F*. *gigantica* and *F*. *hepatica* retrieved from various geographic regions and stored in the GenBank database. Based on haplotype analysis of mitochondrial genes, the snail intermediate host harbours the same *F*. *hepatica* haplotype infecting humans in the studied area. Overall, the haplotype diversity of our sequences compared to reference sequences was lower in *F*. *hepatica* group than *F*. *gigantica* group. This is consistent with a previous report on bovine fascioliasis in Egypt that showed a higher genetic diversity of *F*. *gigantica* than *F*. *hepatica* [14]. In contrast to our results, Amer *et al*. reported a higher haplotype diversity of the mitochondrial NADI gene in *F*. *hepatica* than in *F*. *gigantica* [47]. Also, numerous researchers have formerly described the heterogeneous nature of the *F*. *hepatica* genome [96–98]. This disparity is primarily attributable to differences in the number of both species of flukes characterized in those studies [47].

Noteworthy, our isolates, whether *F*. *hepatica* or *F*. *gigantica* belonged to one or two haplotypes that revealed the low genetic variation in the studied samples. This could be attributed to the small sample size, limited host distribution, and the restricted study area (the area of *Fasciola* outbreak). Therefore, further research is needed on a wide geographic area including different types of animal hosts and different lymnaeid species.

### Limitations and conclusions

In conclusion, the present study reported significant findings regarding the epidemiological overview of fascioliasis in a newly established focus in Upper Egypt. Several factors, such as the relatively small sample size and the lack of data regarding the effects of climate change, seasonality, and the spatial distribution of the snail intermediate host, could limit the scope of the study regarding the transmission pattern of the disease. In addition, the study emphasized the significance of screening asymptomatic relatives in the neighborhood of suspected fascioliasis cases to avoid underestimating the number of human cases and identifying the risk factors of disease transmission. This study is the first molecular evidence of the existence of *R*. *auricularia* in Egypt as the snail intermediate host in a newly emerged endemic area in Assiut Governorate, Upper Egypt, to our knowledge. The findings indicated that the governorate of Assiut can be considered a high-risk area for human populations, and health care policies must take fascioliasis into account. Inferred from the data presented here is the presence of *F*. *hepatica* as the chief *Fasciola* spp. infecting human and snail intermediate host in this new endemic region. Further systematic research is required to clarify the genetic diversity and nationwide distribution of *Fasciola* in different hosts and field-collected snails in Egypt using morphological and molecular approaches. Also, extended studies are required to clarify the faunistic composition of lymnaeid snails in Upper Egypt using multiple nuclear and mitochondrial genes. This study can be viewed as an initial step toward comprehending the epidemiological situation of human fasciolosis and the genetic diversity of the *Fasciola* population in a newly emerged endemic focus in Upper Egypt.

## Supporting information

S1 Fig Neighbor-Joining phylogenetic tree based on the ribosomal ITS-1 analysis of Egyptian isolates of *Fasciola* spp. and other representative isolates in the GenBank database from different localities. The evolutionary distances were computed using the Tamura 3- parameter method. The tree is drawn to scale.
(TIF)

S2 Fig Molecular phylogenetic analysis of *Radix* spp. using the ML method based on the Tamura 3-parameter model. The tree is drawn to scale, with bootstrap value next to branches.
(TIF)

S3 Fig Neighbor-Joining Phylogenetic tree: Showing the evolutionary relationships between Egyptian isolates of *Fasciola* spp. and references sequences based on the mitochonderial NADI gene analysis. The percentage of replicate trees in which the associated taxa clustered together in the bootstrap test (1000 replicates) are shown next to the branches. The tree is drawn to scale.
(TIF)

S4 Fig Neighbor-Joining phylogenetic tree showing the evolutionary relationships between Egyptian isolates of *Fasciola* spp. and references sequences based on the mitochondrial COXI gene analysis using the Tamura 3-parameter method with bootstrap value next to branches (1000 replicates). (TIF)
