# Supplementary figures and images for: New perspectives for fascioliasis in Upper Egypt’s new endemic region: Sociodemographic characteristics and phylogenetic analysis of Fasciola in humans, animals, and lymnaeid vectors
Source: PLoS Negl Trop Dis. 2022 Dec 28;16(12):e0011000. doi: 10.1371/journal.pntd.0011000 (PMC9797099; doi:10.1371/journal.pntd.0011000)

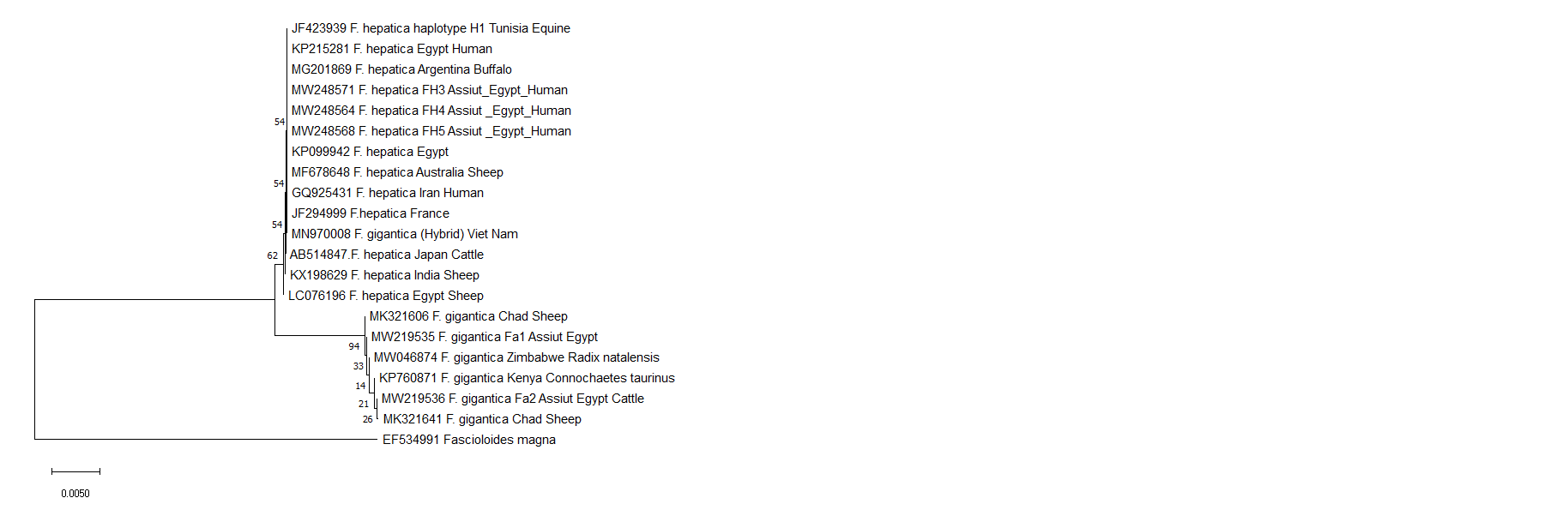

Supplement: S1 Fig — The evolutionary distances were computed using the Tamura 3- parameter method. The tree is drawn to scale. (TIF) [file pntd.0011000.s001.tif]

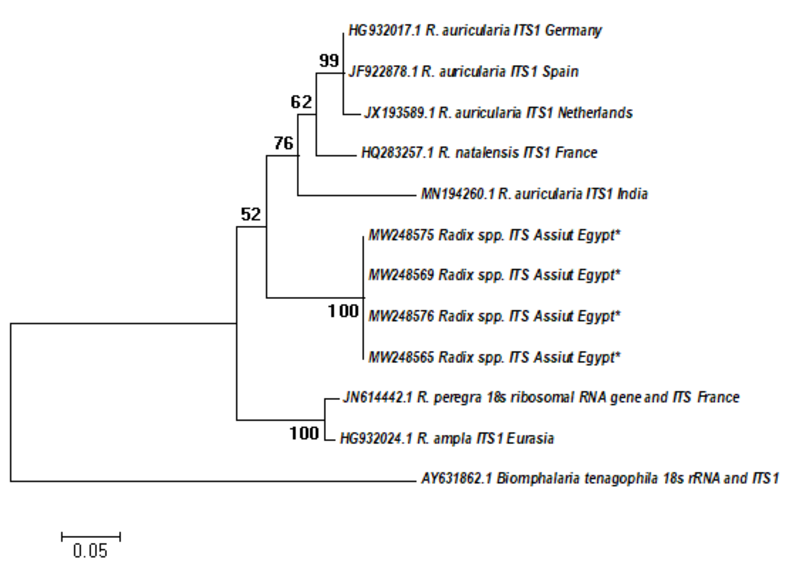

Supplement: S2 Fig — The tree is drawn to scale, with bootstrap value next to branches. (TIF) [file pntd.0011000.s002.tif]

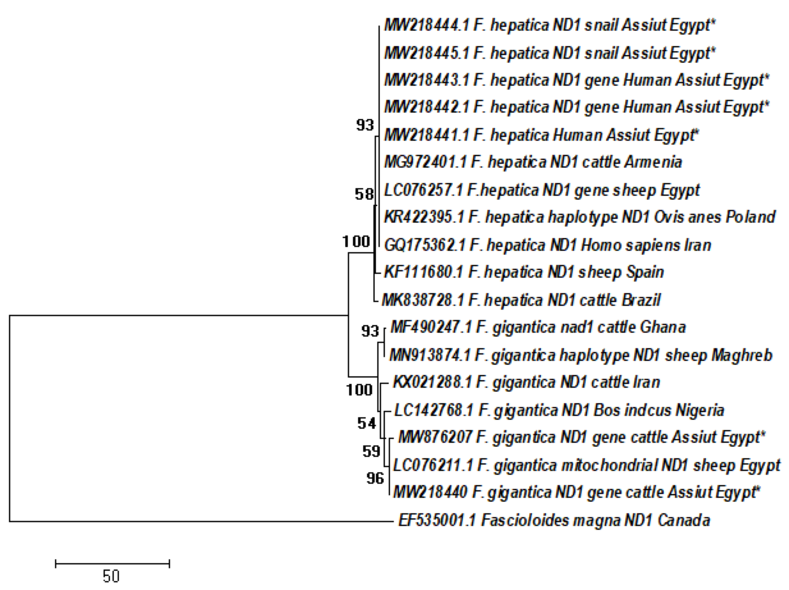

Supplement: S3 Fig — The percentage of replicate trees in which the associated taxa clustered together in the bootstrap test (1000 replicates) are shown next to the branches. The tree is drawn to scale. (TIF) [file pntd.0011000.s003.tif]

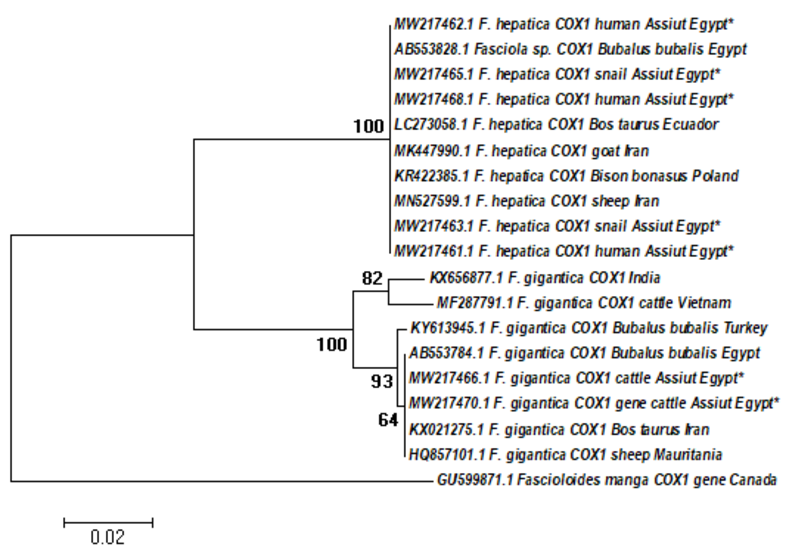

Supplement: S4 Fig — (TIF) [file pntd.0011000.s004.tif]
